# Characterization of *Edwardsiella* Strains Isolated from Fish in The Netherlands (1995–2020) Shows a Relationship Between *Edwardsiella* Species and Source

**DOI:** 10.3390/pathogens15090911

**Published:** 2026-08-29

**Authors:** Janneke Allaart, Andries Kampfraath, Frank Harders, Michal Voorbergen-Laarman, Betty van Gelderen, Belén Fouz, Marc Engelsma

**Affiliations:** 1 Wageningen Bioveterinary Research, 8221 RA Lelystad, The Netherlands; dre.kampfraath@wur.nl (A.K.); frank.harders@wur.nl (F.H.); michal.voorbergen-laarman@wur.nl (M.V.-L.); betty.vangelderen@wur.nl (B.v.G.); marc.engelsma@wur.nl (M.E.); 2 University Institute of Biotechnology and Biomedicine Research (BIOTECMED), Universitat de València, 46100 Burjassot, Spain; belen.fouz@uv.es

**Keywords:** *Edwardsiella*, fish species, MLSA, MALDI-TOF MS

## Abstract

To date, four species of the genus *Edwardsiella*—*E. tarda*, *E. ictaluri*, *E. piscicida*, and *E. anguillarum*—have been identified as the cause of disease and mortality in various fish species. Thirty-six *Edwardsiella* strains isolated from different fish species in the Netherlands were identified using multilocus sequence analysis (MLSA) based on sequence analysis of the genes *gyrB*, *pgi* and *phoU*. In parallel, an *Edwardsiella* matrix-assisted laser desorption ionization–time of flight mass spectrometry (MALDI-TOF MS) database was created by analyzing peptide spectra of a subset of these strains belonging to the four fish pathogenic species. MALDI-TOF MS analysis of the *Edwardsiella* isolates using the newly created database revealed 100% agreement with results of MLSA. Moreover, the presence of each *Edwardsiella* species was associated with specific fish host and source, with *E. piscicida* being the dominant species in Dutch aquaculture. In addition, a novel strain was isolated from electric eel, genetically diverging from the four established species. Whole-genome comparisons of the novel strain with those species showed DNA-DNA hybridization and average nucleotide identity scores suggestive of a new species. In conclusion, both MLSA and MALDI-TOF analysis can be used to identify *Edwardsiella* species. Moreover, it has been observed that different *Edwardsiella* species show a preference for certain hosts or sources.

## 1. Introduction

Bacteria belonging to the genus *Edwardsiella* are considered to be among the most pathogenic bacteria affecting fish. *Edwardsiella* species are Gram-negative, motile, short, rod-shaped, facultative aerobic bacteria responsible for the disease commonly known as ‘fish gangrene’, ‘emphysematous putrefactive disease’ or ‘red disease of eels’, leading to mass mortality in various populations and age groups of fish [1]. In 1965, *Edwardsiella tarda* was the first *Edwardsiella* species identified as a distinct taxon within the family Enterobacteriaceae [2]. *Edwardsiella ictaluri*, causing enteric septicemia in catfish (ESC), was the next species identified in 1976 [3], and in 1980, a third species was identified from birds and reptiles: *Edwardsiella hoshinae* [4].

More recently, sequencing of the genome of six different *E. tarda* strains from different origins demonstrated two genetically distinct, polyphyletic groups [5]. Also, multilocus sequence analysis (MLSA) from twenty-seven *E. tarda* isolates supported the demonstration of two genetically distinct groups [6]. Subsequent genotypic and phenotypic analyses of fifteen of these isolates revealed a novel species within the genus *Edwardsiella*, for which the name *Edwardsiella psicicida* was proposed [7]. Simultaneously, a third variant was isolated from eel and sea bream characterized by phylogenetic analysis [7], as well as in another study from tilapia using repetitive sequence-mediated PCR [8]. A phylogenetic analysis based on the *gyrB* gene confirmed that these isolates belonged to the same subgroup, and the genetic variant was called *E*. *piscicida*-like [9]. In a subsequent study, more strains from the same variant were identified from eel and sea bream, and in addition, the variant was proposed as a new species: *Edwardsiella anguillarum* [10]. Consequently, the *Edwardsiella* genus now consists of five species.

A study by Reichley and co-workers [11] evaluated phenotypic and genotypic analyses, microbial identification systems and molecular confirmatory methods for differentiating between the five *Edwardsiella* species. Phylogenetic analysis revealed that partial 16S rRNA had low discriminatory power. However, single-copy genes *gyrB* (encoding DNA gyrase subunit B) and *sodB* (encoding superoxide dismutase B) displayed high discriminatory power, while in the meantime maintaining high intraspecific similarity between *Edwardsiella* species. Also, the genes *pgi* (encoding glucose-6-phosphate isomerase) and *phoU* (encoding phosphate-specific transport system accessory protein) have a high discriminatory power [12,13]. Therefore, MLSA analyses based on these genes can be used for differentiation between the *Edwardsiella* species.

Matrix-assisted laser desorption ionization–time of flight (MALDI-TOF) is an emerging technology for rapid identification of microorganisms. With this method, bacteria can be identified based on their proteomic mass fingerprint after ionization of a sample and determining the mass-to-charge ratio (*m*/*z*) based on the time of flight. Compared to MLSA, MALDI-TOF strongly reduces the time for identification of isolated strains. At present, only representatives of *E. tarda*, *E. ictaluri* and *E. hoshinae* are present in the Bruker spectrum database. The Bruker MALDI-TOF method correctly identified all *E. tarda*, *E. ictaluri*, and *E. hoshinae* isolates, while other phenotypical tests failed to 100% discriminate between the three *Edwardsiella* species [11]. In the study, *E. piscicida* and *E. anguillarum* isolates were also identified as *E*. *tarda*; however, unique species-specific peptide mass peaks were observed in the spectral profiles for *E. anguillarum*, *E. piscicida*, and *E. tarda*, respectively, suggesting that MALDI-TOF analysis can offer rapid, reliable identification of the five *Edwardsiella* species [11].

Over the past few decades, *Edwardsiella* species have been regularly isolated from diseased fish in the Netherlands (unpublished data), making it a reoccurring problem in aquaculture as well as in wild and ornamental fish. *Edwardsiella* bacteria were isolated mainly from European eel, which is an important fish species in Dutch aquaculture. Furthermore, the bacterium was detected in turbot, common sole, catfish and several ornamental fish species. In the diagnostic process, all strains are identified at genus or species level by biochemical tests or MALDI-TOF analysis.

In order to explore the presence of the different *Edwardsiella* species in Dutch fish and to validate reliable detection of the four fish-pathogenic *Edwardsiella* species by MALDI-TOF, a collection of thirty-six *Edwardsiella* strains isolated from diseased fish (different sources) and one environmental strain (from water) were identified to species level based on an MLSA of the *gyrB*, *pgi* and *phoU* genes. An *Edwardsiella* species main spectrum projection (MSP) library was created using MALDI-TOF and a selection of the typed strains. Subsequently, the new custom MALDI-TOF database was validated using strains belonging to the four different *Edwardsiella* species.

## 2. Materials and Methods

### 2.1. Edwardsiella *spp.* Isolates and Culture Conditions

Thirty-six strains from diseased fish and one environmental strain (from water) collected from diagnostic samples from the Netherlands over the period 1995–2020 were used in this study (Table 1, further background information on clinical signs is provided in Supplemental Table S1). The strains were previously identified as *Edwardsiella* spp. by biochemical properties or MALDI-TOF analysis. Isolates were stored at −80 °C using glycerol (30%) stocks. Isolates were thawed and grown on Heart Infusion supplemented with 5% sheep blood (HIS) agar plates (in house WBVR) under aerobic conditions for 48 h at 22 °C. Subsequently, the strains were subcultured for another 48 h at 22 °C before using the strains for further analysis.

### 2.2. Lysate Preparation, PCR and Sequencing

A full 10 µL inoculation loop of bacterial colonies from the agar plate (pure culture) was suspended in 500 µL distilled water. The suspension was heated for 10 min at 98 °C and subsequently 1:10 diluted and frozen at −20 °C until further processing. Amplifications were performed on a ProFlex PCR System (Applied Biosystems, Waltham, MA, USA). Primers used for PCR amplification and sequencing of the *pgi*, *phoU* and *gyrB* genes, originally described by Griffin and co-workers (2013; 2014), are listed in Table 2.

For *pgi*, *phoU*, *GyrB-1* and *GyrB-2,* amplification reactions (50 µL) were performed using 31.5 µL Super-Q water, 5 µL PCR buffer (10×), 5 µL MgCl_2_, 1 µL deoxynucleoside triphosphate mix (2.5 mM each), 0.2 µM of each primer, 0.5 µL AmpilTaq Gold polymerase (5 U/µL, Applied Biosystems) and 5 µL template DNA with the following cycling conditions: 3 min of denaturation at 94 °C; 45 cycles of 30 s at 94 °C, 30 s at 52 °C, and 2 m at 68 °C; and 7 min of extension at 68 °C.

For *GyrB-3* and *GyrB-4,* amplification reactions (50 µL) were performed using 18.5 µL Super-Q water, 10 µL SuperFi-Buffer (5×), 10 µL SuperFi-GC-enhancer (5×), 1 µL deoxynucleoside triphosphate mix (2.5 mM each), 0.5 µM of each primer, 0.5 µL Platina SuperFi DNA polymerase (2 U/µL, Invitrogen, Waltham, MA, USA) and 5 µL template DNA with the following cycling conditions: 30 s of denaturation at 98 °C; 35 cycles of 10 s at 98 °C, 10 s at 60 °C, and 1 m at 72 °C; and 5 min of extension at 72 °C.

Amplicons were visualized using the E-gel system (Invitrogen) with a 2% agarose E-gel. The remaining PCR product was purified using QIAquick columns (Qiagen, Hilden, Germany). Purified PCR products were amplified in a sequence PCR using 2 µL Big Dye Reactionmix (BigDye v 3.1, Applied Biosystems), 1 µL Sequence-buffer (5×) (BigDye v 1.1, Applied Biosystems), 1 µL Super-Q water, 0.4 µM of each primer (Table 2) and 4 µL purified PCR product. Sequence PCR products were subsequently purified using Sephadex™ G-50 M DNA Grade (Cytiva, Marlborough, MA, USA) in 96-well AcroPrep™ filter plates (Cytiva). Sequencing was performed on the SeqStudio Genetic Analyzer (Applied Biosystems).

### 2.3. Phylogenetic Analysis of Isolates

The raw sequence data were analyzed using Sequencher 5.4.6 (Gene Codes Corporation, Ann Arbor, MI, USA). The processed consensus sequences were aligned using MAFFT v7.475 [14] and the phylogeny of the different genes was reconstructed using maximum likelihood (ML) analysis with IQ-TREE software v2.0.3 and 1000 bootstrap replicates [15] and subsequently visualized using the R package ggtree [16]. For the concatenation of the *gyrB*, *pgi* and *phoU* gene sequences, the R package concatipede was used [17]. As a backbone for the phylogenetic tree, additional nucleotide sequences from *Edwardsiella* species were downloaded from NCBI GenBank [18]. The sequences obtained in this study are available under GenBank accession numbers from PZ797225 to PZ797369.

### 2.4. MALDI-TOF Database Creation and Validation

A commercial MALDI-TOF mass spectrometer (Bruker MALDI-TOF Biotyper Sirius GP system, Bruker, Bremen, Germany) was used for generation of peptide mass spectral profiles (MSP).

Based on the results of the MLSA, bacterial colonies from 1–5 strains per species plus an additional *E. anguillarum* reference strain (Ea BF1) were picked from the agar plate and suspended in 300 µL distilled water. Then, 900 µL ethanol (100%) was added and the suspension was mixed well. The suspension was centrifuged at 18.407 rcf and the supernatant was discarded. The centrifugation step was repeated once more and the pellet was dried for 5 min. Then 25 µL of 70% formic acid was added, mixed well and left for 3 min. After this, 25 µL acetonitrile was added and mixed well. The suspension was centrifuged at 18.407 rcf and the supernatant was used for further investigation.

The supernatant was applied to eight spots on the MALDI-TOF target plate (1 µL per spot) and overlaid with 1 µL α-cyano-4-hydroxycinnamic acid (HCCA) matrix solution according to the manufacturer’s recommended protocol. Each spot was measured three times. Mass accuracy was calibrated using the Bruker Bacterial Test Standard.

The spectra were captured in positive linear mode in a mass range of 2 to 20 kDa with a laser frequency of 200 Hz (IS1, 20 kV; IS2, 18.3 kV; lens, 6 kV; extraction delay time, 390 ns). Spectra were acquired in automatic mode by accumulating a maximum of 240 profiles (6 × 40 laser shots from different positions of the target spot).

The peptide spectra were collected and analyzed using flexControl 3.4 software (Bruker Daltonics). Flatline spectra and spectra with other anomalies were removed. Furthermore, the spectra were analyzed for the presence or absence of the unique species-specific peptide mass peaks of *E. anguillarum*, *E. piscicida*, *E. tarda* and *E. ictaluri* [11].

A minimum of 20 spectra were used for MSP creation. The controlled spectra were loaded to the Maldi Biotyper Compass HT software v1.4 (Industry version, Bruker Daltonics) and MSPs were created. The new MSPs were added to a separated *Edwardsiella* database.

After creating the database, bacterial colonies from the remaining strains were picked from the agar plate and applied to one spot on the MALDI-TOF target plate and overlaid with 1 µL α-cyano-4-hydroxycinnamic acid (HCCA) matrix solution. The strains were identified by MALDI-TOF and the results were compared with the obtained genetic data. A score value > 2.0 indicates a high probability of species-level identification; a score value > 1.7 suggests genus-level identification, while score values < 1.7 are generally considered unreliable for identification. Besides a score value, a consistency value is generated. Category A indicates high confidence, category B indicates moderate confidence, and category C means that the result is unreliable.

### 2.5. Whole-Genome Sequencing, Characterization and Comparison of Edwardsiella spp.

The initial results from the Sanger sequencing showed a divergence of strain WBVR15015646 from the known *Edwardsiella* species. To explore this further, it was decided to fully sequence the strain. For whole-genome sequencing of strain WBVR15015646, genomic DNA was extracted with the Qiagen blood & tissue kit following the manufacturer’s instructions, and both long- and short-read sequencing was done. For the short-read library preparation, the Illumina DNA Prep kit (Illumina, Inc., San Diego, CA, USA) was used with 50 ng DNA input according to the manufacturer’s instructions. The recommended number of PCR amplification cycles was applied. Library quality was assessed using the Quant-iT™ dsDNA High Sensitivity Assay Kit (Thermo Fisher Scientific, Waltham, MA, USA) and the Agilent TapeStation 2200 (Agilent Technologies, Inc., Santa Clara, CA, USA) with the High Sensitivity D1000 ScreenTape assay. The library met the required quality criteria for DNA concentration and average fragment size. Short-read sequencing was performed on an Illumina NextSeq 2000 platform, with output targeted to achieve an average genome coverage of approximately 125×, assuming a genome size of 4 Mb. For long-read sequencing, libraries were prepared from 400 ng genomic DNA using the Oxford Nanopore Native Barcoding Kit 96 V14 (SQK-NBD114.96, Oxford Nanopore Technologies, Oxford, United Kingdom) according to the manufacturer’s instructions, and sequenced on an Oxford Nanopore PromethION using an R10.4.1 flow cell. Basecalling was performed with Dorado v 0.9.0 (Oxford Nanopore Technologies, Oxford, United Kingdom) with a highly accurate basecalling model (dna_r10.4.1_e8.2_400bps_hac@v5.0.0).

Sequencing data were processed using an in-house bioinformatics pipeline. Read quality was assessed with FastQC v0.12.1. Hybrid assembly was performed with Hybracter v0.7.3 (hybracter hybrid) with a specified chromosome size of 4 Mb. Within this workflow, long reads were filtered and subsampled with Filtlong (default target depth 100×) and adapter-trimmed with Porechop_ABI, while short reads were quality-controlled with fastp. Long reads were assembled with Flye, followed by targeted plasmid recovery with Plassembler, long-read polishing with Medaka, reorientation of circularized chromosomes to begin with the dnaA gene using Dnaapler (v 1.3.0.) [19], and short-read polishing with Polypolish and pypolca. Each polishing round was scored with ALE, and the final assembly was selected according to Hybracter’s default selection logic. Sequences shorter than 200 bp (*n* = 10) were excluded, consistent with the standard contig length cutoff used for submissions to EMBL-EBI (project accession number PRJEB121486). Genome annotation was performed with Bakta (v1.11.4; database v6.0, full) [20], and assembly quality was evaluated using CheckM2 v1.1.0 with the corresponding reference database.

To compare the *Edwardsiella* species, the following five *Edwardsiella* reference genomes were collected from GenBank: *E. anguillarum* ET080813 (GenBank: GCA_000264765.2), *E. hoshinae* (GenBank: GCA_016026395.1), *E. piscicida* (GenBank: GCA_021733145.1), *E. ictaluri* (GenBank: GCA_003074995.2), and *E. tarda* (GenBank: GCA_019933175.1). Assemblies were reoriented with Dnaapler (v1.3.0.). Alignments of the main chromosome were made with progressiveMauve (build date 25 Feb 2015) [21] and displayed using pyGenomeViz (v1.6.1) [22].

DNA-DNA hybridization (DDH) values were calculated in silico using the genome-to-genome distance calculator webtool (GGDC 3.0) [23]. Average nucleotide identity (ANI) values were calculated using FastANI (version 1.34) [24].

### 2.6. Biochemical Analysis of Edwardsiella spp.

To compare the biochemical properties of strain WBVR15015646 with the biochemical properties of the four different *Edwardsiella* species pathogenic to fish, a selection of Dutch isolates containing all different species and the *E. anguillarum* reference strain (Ea BF1) were used for biochemical analysis. The commercial API 20E system (bioMérieux, Marcy-l’Étoile, France) was used in accordance with the manufacturer’s instructions. Briefly, bacterial colonies were picked from HIS agar, and API 20E strips were inoculated and incubated for 24–48 h at 37 °C. All reagents were added, a seven-digit profile number was generated, and the results of the biochemical tests were used for comparison between the species.

## 3. Results

### 3.1. Genetic Identification

**Figure 1:**
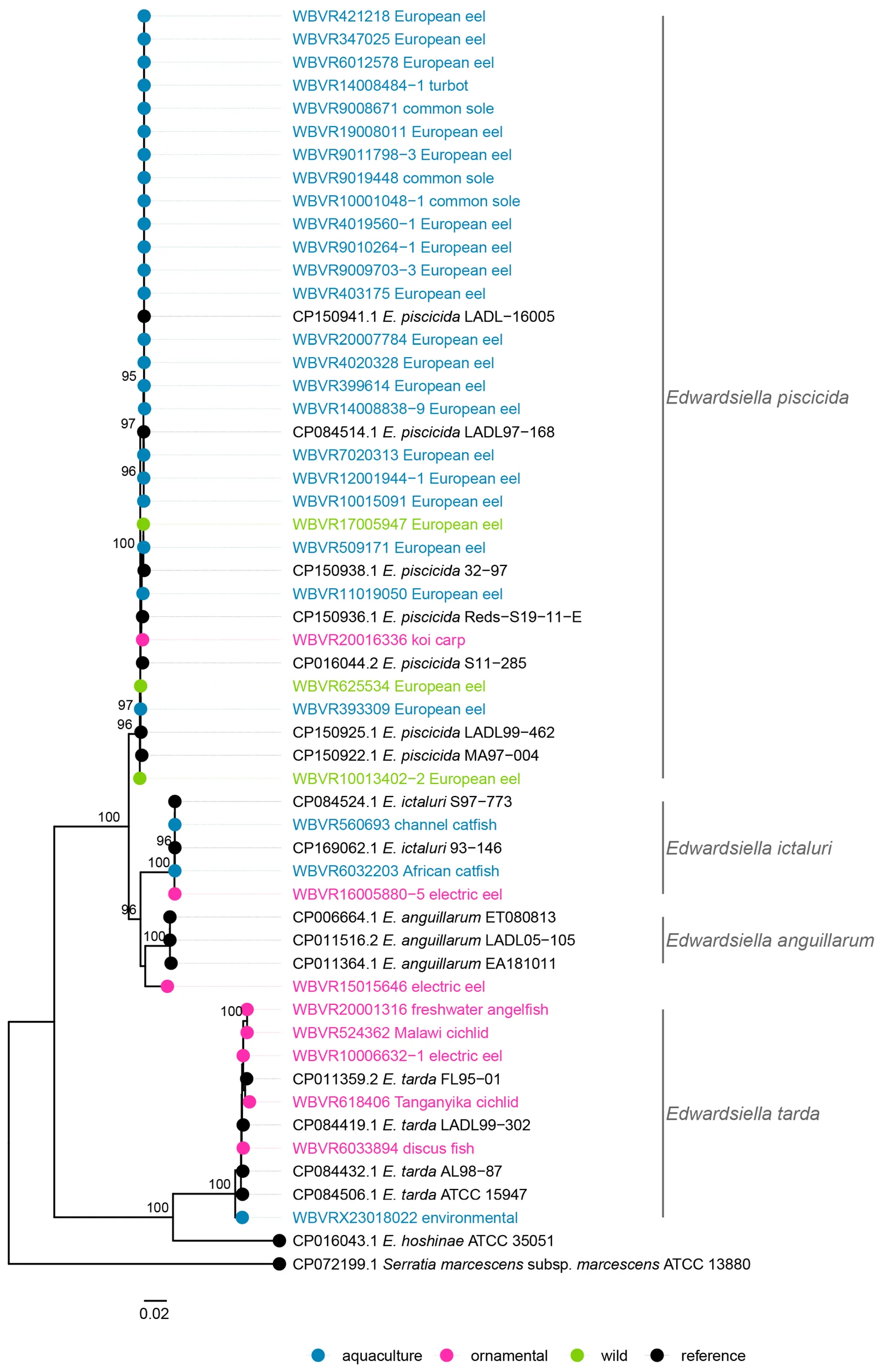
Phylogenetic relationship of *Edwardsiella* species based on the MLSA of the concatenated *gyrB*, *pgi* and *phoU* gene sequences. Reference strains with their GenBank numbers are included (black). The strains characterized in this study are color-coded by origin: cultured (blue), ornamental (magenta) and wild fish (green). IQ-TREE ultrafast bootstrap (UFBoot) values ≥ 95% are indicated at the nodes. *Serratia marcescens* subsp. *marcescens* is used as the outgroup.

Based on the genetic information from the MLSA, all strains could be identified as belonging to one of the described *Edwardsiella* species (Figure 1), with the exception of one strain isolated from an electric eel which was divergent from the other strains.

The *Edwardsiella* species largely corresponded to the source: *E*. *piscicida* was mainly isolated from fish from aquaculture systems including eel, turbot and common sole. Furthermore, *E*. *piscicida* was isolated from wild eel and koi carp. *E*. *tarda* was only isolated from ornamental fish from zoos and traders of (sub)tropical fish and from a water sample from a catfish farm. *E. ictaluri* was recovered from both catfish and electric eel. *E*. *anguillarum* was not isolated from fish in the Netherlands during the study period.

### 3.2. MALDI-TOF: MSP-Profiles

For *E. tarda* and *E. piscicida*, the MSP profiles created from one and five strains respectively were added to the custom *Edwardsiella* database. For *E. ictaluri* there were three strains isolated from the Netherlands added to the custom *Edwardsiella* database. For *E. anguillarum* one reference strain was available and added to the database. Strain WBVR15015646 isolated from electric eel diverged from the known *Edwardsiella* species in both the MLSA analyses and the MALDI-TOF MS analysis. It was therefore added with its own MSP profile in the custom database. Five *E. piscicida* strains, five *E. tarda* strains and three *E. ictulari* strains were used to validate the custom database. If the strain was present in the custom database, the result of the corresponding strain was removed from the validation results. For *E. anguillarum* and the novel *Edwardsiella* sp., only one strain was available, limiting the validation. Table 3 provides an overview of the strains used to create a custom MALDI-TOF database and to validate this custom database.

Strains were tested twice, first using the Bruker MALDI-TOF database and subsequently using the Bruker MALDI-TOF database combined with the newly created *Edwardsiella* MALDI-TOF MS database. Using the standard Bruker database, the strains identified by MLSA as *E. tarda* and *E. ictaluri* were correctly identified (Table 4). In general, strains identified by MLSA as *E. piscicida*, *E. anguillarum* and the strain not belonging to any of the well-described *Edwardsiella* species were identified as *Edwardsiella* (score > 1.7) using the standard Bruker database, but could not be identified to species level (score > 2.0). Only one *E. piscicida* strain had a score value of 2.0 with a high consistency score (A), but the strain was misidentified as *E. tarda*. When testing the strains using the combined databases all strains were identified in accordance with their MLSA typing. Only the *E. anguillarum* strain still had a medium consistency score (B), probably because the entry is limited to the single *E. anguillarum* strain present in the database. The custom *Edwardsiella* spectrum database is available on request from the authors.

Spectra of the strains selected for the MALDI-TOF database were also analyzed for the presence of the earlier-identified unique species-specific peptide mass peaks of *E. anguillarum*, *E. piscicida*, *E. tarda* and *E. ictaluri* [11]. However, these mass peaks were detected only in a subset of the analyzed strains.

### 3.3. Charaterisation of Edwardsiella sp.

The genome of *Edwardsiella* sp. was assembled and compared to reference strains of the five known *Edwardsiella* species. The first *Edwardsiella* sp. assembly contained 18 contigs; these were annotated with Bakta. Examination of the DNApler output identified origins of replication in four contigs, three of which were circular; these three circular contigs were treated as the draft genome. Among the remaining five non-circular contigs, one encoded a repA gene and three contained an RNAI locus, suggesting the possible presence of an additional plasmid that was not sequenced to sufficient depth to allow complete reconstruction. As plasmid characterization was not the primary focus of this study, these contigs were not analyzed further.

**Figure 2:**
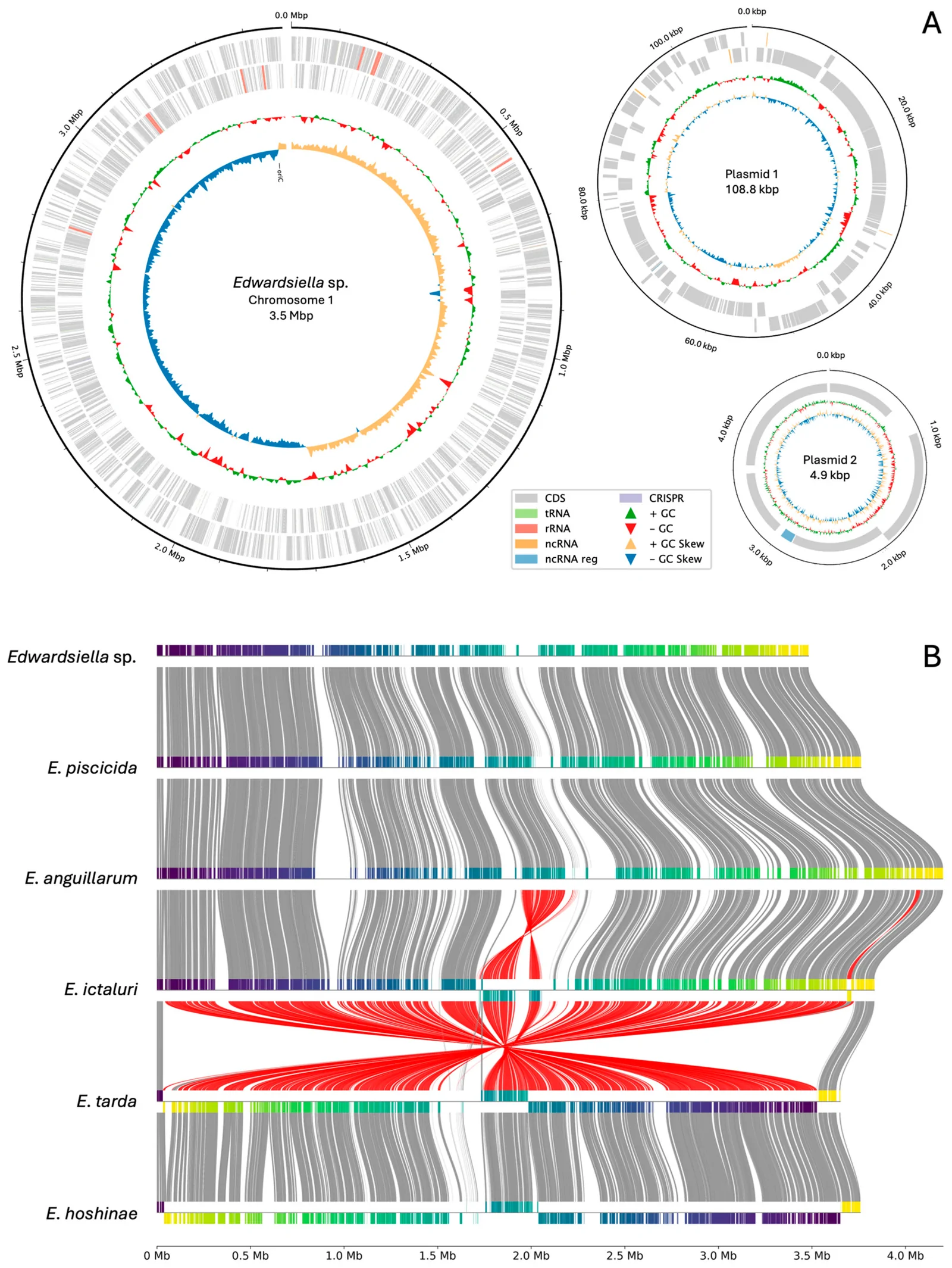
(**A**) Circular representation of the *Edwardsiella* sp. genome based on Bakta annotation output. Left, chromosome; right, plasmids. From the outer to inner rings, the plot displays (1) genome coordinates; (2–3) predicted coding and non-coding features on the forward and reverse strands, including CDS, rRNA, tRNA, ncRNA and ncRNA reg; (4) GC content relative to the genome-wide mean; and (5) GC skew, indicating strand asymmetry and putative replication origin and terminus. (**B**) Progressive Mauve alignment of *Edwardsiella* spp. genomes showing locally collinear blocks (LCBs). Each horizontal panel represents one genome; colored LCBs denote homologous regions. Blocks above the centerline indicate forward orientation relative to the reference; those below indicate reverse complement orientation. Inversions are highlighted in red.

The three remaining contigs, consisting of one main chromosome and two plasmids, were re-annotated with Bakta individually (Figure 2A). With a size of 3.5 Mbp, this is the smallest *Edwardsiella* genome, containing the fewest coding sequences (Supplementary Table S2). While *Edwardsiella* sp. is most closely related to *E. anguillarum* and *E. piscicida*, the in silico DDH values below 70% and ANI scores below 95%, the generally accepted thresholds for species-level delineation [23,24], support its distinction from these species (Table 5). In addition, structurally the genome is also closest to *E. piscicida* (Figure 2B). While large genomic rearrangements can be seen between *E. tarda* and *E. hoshinae* versus the other *Edwardsiella* species, the most notable difference between *Edwardsiella* sp. and *E. piscicida* is the reduction in size, most likely due to multiple smaller deletions.

### 3.4. Biochemical Analysis

The biochemical properties of strain WBVR15015646 were most similar to *E. ictaluri* and very different from *E. piscicida*, *E. anguillarum* and *E. tarda* (Supplementary Table S3). The API 20E system could not distinguish between the latter three species, which is in line with previous findings [11].

## 4. Discussion

To investigate the occurrence and severity of fish diseases caused by *Edwardsiella* species, accurate identification of the causative agent is of prime importance. Using genotypic analysis such as MLSA or whole-genome analysis, the five different *Edwardsiella* species can be readily distinguished [5,6,9,11]. However, both techniques are still relatively expensive and labor-intensive. Matrix-assisted laser desorption ionization–time of flight (MALDI-TOF) offers a rapid and cost-effective method for the identification of bacteria. However, the accuracy of the method depends on the completeness of the database, and the identification of the *Edwardsiella* species is hampered by the absence of several species from this genus in the standard database provided by the manufacturer. In an earlier study, discriminatory peaks for each *Edwardsiella* species were described [11]. However, analysis of the mass spectra in our study revealed that these unique, species-specific peptide mass peaks were not consistently present in all spectra. Nevertheless, using a newly created MALDI-TOF database with spectra of the four described fish-pathogenic *Edwardsiella* species in addition to the standard Brüker database, all strains could be identified to species level. The consistency value was high for all species except for *E. anguillarum* (medium). Including additional *E. anguillarum* strains will most likely increase the consistency value. Furthermore, for full validation of the database, testing of additional *E. anguillarum* strains is necessary.

With regard to MLSA of the strains from Dutch aquaculture, ornamental fish, wild fish and the environment, 27 were identified as *E. piscicida*, six as *E. tarda*, three as *E. ictaluri* and one as a potential novel *Edwardsiella* species. The species largely corresponds to the source: *E*. *piscicida* was isolated from cultured fish, wild eel and koi carp while *E*. *tarda* was isolated from ornamental fish from zoos and traders of (sub)tropical fish. These results may indicate a relationship between species identity and the source of isolation.

The fact that *E. piscicida* was the dominant species in Dutch aquaculture in this study is consistent with previous research, in which MLSA-typed strains—isolated from diseased eel, turbot and whitefish from aquaculture in Norway, the UK and Finland—that had been identified as *E. tarda* were reclassified as belonging to the species *E. piscicida* [6,9,10,25]. Publications prior to 2012 on *E. tarda* should be regarded with some reservation as this could well be *E. piscicida*. Therefore, *E. piscicida* seems to be the major *Edwardsiella* species in aquaculture in North-Western Europe.

In our study *E. tarda* was only isolated from ornamental fish and from a water sample from a catfish farm where no clinical signs were observed. There are very few studies that describe the isolation of *E. tarda* from ornamental fish species. *E*. *tarda* strains have been recovered from Asian swamp eel [26] as well as from Oscar fish [27]. Besides from ornamental fish species, *E. tarda* has been isolated from (Nile) tilapia [9,28,29], hybrid striped bass [9] and catfish [9,27,30], farmed in regions where relatively high seasonal water temperatures can occur (25–30 °C) compared with Dutch aquaculture in indoor recirculation systems (18–25 °C). Therefore, a preference of *E. piscicida* for low water temperatures and of *E. tarda* for high water temperatures—rather than a preference for a specific host—could therefore explain why, in our study, *E. piscicida* was isolated from diseased fish from aquaculture sources, whereas *E. tarda* was isolated exclusively from ornamental fish. However, in vitro data do not fully support this hypothesis; although *E. piscicida* may grow at a lower temperatures than *E. tarda*, *E. piscicida* has also been shown to grow at an in vitro culture temperature of 37 °C, which is much higher than the water temperature in fish husbandry systems [6,10,25,26,31]. Therefore, environmental factors other than water temperature may contribute to the susceptibility of fish to certain *Edwardsiella* species.

*E. tarda* has zoonotic potential [9,10], whereas *E. piscicida* has thus far not been isolated from humans. The fact that only *E. piscicida* was isolated from fish from Dutch aquaculture and from wild eel from the Netherlands may explain why human cases of edwardsiellosis (which are generally attributed to *E. tarda*) are rare in the northern hemisphere and mainly occur in (tropical) regions [32]. *E. tarda*-infected ornamental fish in the northern hemisphere, imported from these tropical regions, potentially represent a zoonotic risk. However, case studies from ornamental fish are rare and only one case study on human edwardsiellosis traced to ornamental fish has been published so far [33].

*E. anguillarum* has not been isolated from fish in the Netherlands. The bacterium has not been isolated from the North of Europe so far. *E*. *ictaluri* was only isolated from catfish, the most frequent host, and electric eel [9,10]. The fact that the three strains isolated from the electric eels were genetically very different from each other shows that the electric eel is susceptible to several *Edwardsiella* species and may therefore serve as a “mixing vessel” for the emergence of new *Edwardsiella* species.

One specific strain isolated from an electric eel (WBVR15015646) diverged from the previously described species, both genetically and in its protein spectra. The in silico DDH and ANI scores showed a close relationship to *E. anguillarum* and *E. piscicida*, also reflected in the phylogenetic tree based on the concatenated *gyrB*, *pgi* and *phoU* gene sequences. Structurally, based on the whole genome, the strain is closest to *E. piscicida*. However, the genome was smaller than the genome of the other species and differed sufficiently to be considered a separate species. Moreover, the biochemical properties were very different from those of *E. piscicida* and *E. anguillarum* strains. The strain originated from the electric organs of an electric eel at a Dutch zoo, where a high mortality rate among the recently imported electric eels was reported. It would be interesting to screen more *Edwardsiella* isolates from imported ornamental fish with MALDI-TOF to determine whether comparable strains are present. Subsequent genetic analysis could then confirm the existence of a sixth *Edwardsiella* species.

## 5. Conclusions

In conclusion, in the recent Dutch aquaculture the *Edwardsiella* species present seems to be limited to *E. piscicida*, while in (sub)tropical ornamental fish *E. tarda* was primarily observed. MALDI-TOF analysis can be used to rapidly differentiate species within the genus for further research on host and environmental preferences as well as zoonotic risk of species. Adding additional *E. anguillarum* strains to the newly developed database will increase the consistency value of the measurements. Furthermore, strain WBVR15015646 isolated from electric eel diverged from the currently known *Edwardsiella* species. Collecting similar strains by typing isolates from (sub)tropical fish could support the description of this possible new *Edwardsiella* species.

## Figures and Tables

**Table 1:** Overview of *Edwardsiella* isolates used in this study.

Isolate ID	Year	Host	Scientific Name	Source
WBVR4020328	1995	European eel	*Anguilla anguilla*	aquaculture
WBVR4019560	1996	European eel	*Anguilla anguilla*	aquaculture
WBVR6012578	1996	European eel	*Anguilla anguilla*	aquaculture
WBVR403175	1996	European eel	*Anguilla anguilla*	aquaculture
WBVR421218	1997	European eel	*Anguilla anguilla*	aquaculture
WBVR509171	1998	European eel	*Anguilla anguilla*	aquaculture
WBVR524362	1999	Malawi cichlid	unknown	ornamental
WBVR560693	2000	channel catfish	*Ictalurus punctatus*	aquaculture
WBVR618406	2002	Tanganyika cichlid	*Thorichthys meeki*	ornamental
WBVR625534	2003	European eel	*Anguilla anguilla*	wild
WBVR4020328	2004	European eel	*Anguilla anguilla*	aquaculture
WBVR4019560	2004	European eel	*Anguilla anguilla*	aquaculture
WBVR6012578	2006	European eel	*Anguilla anguilla*	aquaculture
WBVR6032203	2006	African catfish	*Clarias gariepinus*	aquaculture
WBVR6033894	2006	discus fish	*Symphysodon discus*	ornamental
WBVR7020313	2007	European eel	*Anguilla anguilla*	aquaculture
WBVR9008671	2009	common sole	*Solea solea*	aquaculture
WBVR9009703	2009	European eel	*Anguilla anguilla*	aquaculture
WBVR9010264	2009	European eel	*Anguilla anguilla*	aquaculture
WBVR9011798	2009	European eel	*Anguilla anguilla*	aquaculture
WBVR9019448	2009	common sole	*Solea solea*	aquaculture
WBVR10001048	2010	common sole	*Solea solea*	aquaculture
WBVR10006632	2010	electric eel	*Electrophorus electricus*	ornamental
WBVR10013402	2010	European eel	*Anguilla anguilla*	wild
WBVR10015091	2010	European eel	*Anguilla anguilla*	aquaculture
WBVR11019050	2011	European eel	*Anguilla anguilla*	aquaculture
WBVR12001944	2012	European eel	*Anguilla anguilla*	aquaculture
WBVR14008484	2014	turbot	*Scophthalmus maximus*	aquaculture
WBVR14008838	2014	European eel	*Anguilla anguilla*	aquaculture
WBVR15015646	2015	electric eel	*Electrophorus electricus*	ornamental
WBVR16005880	2016	electric eel	*Electrophorus electricus*	ornamental
WBVR17005947	2017	European eel	*Anguilla anguilla*	wild
WBVR19008011	2019	European eel	*Anguilla anguilla*	aquaculture
WBVR20001316	2020	freshwater angelfish	*Pterophyllum scalare*	ornamental
WBVR20007784	2020	European eel	*Anguilla anguilla*	aquaculture
WBVR20016336	2020	koi carp	*Cyprinus carpio*	ornamental
WBVRX23018022	2023	N.A. *	N.A.	aquaculture

* N.A: from water from RAS system with African catfish (*Clarias gariepinus*).

**Table 2:** Primers used in the current study.

Target	Primer	Sequence (5′–3′)	Reference
*GyrB-1*	GyrB630F	GGATAACGCGATTGACGAAG	[9]
	GyrB1245R	ATCRTCYTTCATGGTCGARA	[9]
*GyrB-2*	GyrB2198F	TAAAGACGATGAGGCGATGG	[9]
	GyrB2540R	GCCGTGARCAAARTCRAA	[9]
*GyrB-3*	GyrB872F	CMCTGTCYGARAAGYTGGAR	[9]
	GyrB1949R	GGAGAGCATCTTGTCGAAGC	[9]
*GyrB-4*	GyrB1425F	ATGACCCGTACGCTGAACA	[9]
	GyrB2540R	GCCGTGARCAAARTCRAA	[9]
*Pgi*	Pgi-F	TGCCGACCGTTTCTCTAAGT	[8]
	Pgi-R	GACCCAGTCCCAGAACTCAA	[8]
*PhoU*	Pho-F	ATATCCGCACCCAGGTAATG	[8]
	Pho-R	TGTCAGCAGCTGTTCCAGAT	[8]

**Table 3:** Strains of *Edwardsiella* used for creation of spectra and for validation.

Isolate	Host	Source	Species	MALDI-TOF Database	Validation Strain
WBVR10001048	common sole	aquaculture	*E. piscicida*	X *	
WBVR10013402	European eel	wild	*E. piscicida*	X	
WBVR14008484	turbot	aquaculture	*E. piscicida*	X	
WBVR20007784	European eel	aquaculture	*E. piscicida*	X	
WBVR20016336	koi carp	ornamental	*E. piscicida*	X	
WBVR625534	European eel	wild	*E. piscicida*		X **
WBVR9008671	common sole	aquaculture	*E. piscicida*		X
WBVR9019448	common sole	aquaculture	*E. piscicida*		X
WBVR12001944	European eel	aquaculture	*E. piscicida*		X
WBVR17005947	European eel	wild	*E. piscicida*		X
WBVR10006632	electric eel	ornamental	*E. tarda*	X	
WBVR618406	Tanganyika cichlid	ornamental	*E. tarda*		X
WBVR6033894	discus fish	ornamental	*E. tarda*		X
WBVR20001316	freshwater angelfish	ornamental	*E. tarda*		X
WBVR524362	Malawi cichlid	ornamental	*E. tarda*		X
WBVRX23018022	n.a.	aquaculture	*E. tarda*		X
Ea BF1	eel	aquaculture	*E. anguillarum*	X	X
WBVR15015646	electric eel	ornamental	*Edwardsiella* sp.	X	X
WBVR560693	channel catfish	aquaculture	*E. ictaluri*	X	X
WBVR6032203	African catfish	aquaculture	*E. ictaluri*	X	X
WBVR16005880	electric eel	ornamental	*E. ictaluri*	X	X

* strain included in the MALDI-TOF database; ** strain used for validation of the database.

**Table 4:** MALDI-TOF scores, species identified, average score and consistency for the standard Bruker database and the Bruker database combined with the custom *Edwardsiella* database.

		Brüker Database						Combined Databases					
Species	No.	Score Value			Species Identified	Average +/− SD	Consistency **	Score Value			Species Identified	Average +/− SD	Consistency
		>2.0	>1.7	<1.7				>2.0	>1.7	<1.7			
*E. tarda*	5	5			*E. tarda*	2.42 +/−0.07	A	5			*E. tarda*	2.26 +/−0.09	A
*E. piscicida*	5	1 *	4		*E. tarda*/ *E. ictaluri*	1.88 +/−0.10	A/ B	5			*E. piscicida*	2.38 +/−0.16	A
*E. anguillarum*	1		1		*E. tarda*	1.88	B	1			*E. anguillarum*	2.38	B
*E. ictaluri*	5	5			*E. ictaluri*	2.18 +/−0.10	A	5			*E. ictaluri*	2.23 +/−0.07	A
*Edwardsiella* sp.	1		1		*E. hoshinae*	1.77	C	1			*Edwardsiella* sp.	2.59	A

* *E. tarda*, score 2.0, consistency A; ** A = high confidence; B = moderate confidence; C = unreliable.

**Table 5:** Cross-table of in silico DNA-DNA hybridization (DDH) values and average nucleotide identity (ANI) values between *Edwardsiella* sp. and references genomes of the five described *Edwardsiella* species. In bold, on the cross-table diagonal, are the homologous reference comparisons.

		ANI					
	Strain	*Edwardsiella* sp.	*E. anguillarum*	*E. piscicida*	*E. ictaluri*	*E. tarda*	*E. hoshinae*
in silico DDH	*Edwardsiella* sp.	**100**	94.4	94.3	92.4	84.1	83.5
	*E. anguillarum*	58.1	**100**	95.0	92.9	84.4	83.6
	*E. piscicida*	57.1	59.9	**100**	92.5	84.1	83.6
	*E. ictaluri*	48.3	49.7	48.5	**100**	83.6	82.9
	*E. tarda*	26.1	25.9	25.6	24.9	**100**	88.8
	*E. hoshinae*	25.0	24.8	24.8	24.2	34.8	**100**

## Data Availability

The GenBank accession numbers of DNA sequence data reported in this study are PZ797225 to PZ797369. The sequencing dataset on *Edwardsiella* sp. WBVR15015646 generated for this study has been deposited in the European Nucleotide Archive (ENA) at EMBL-EBI under the project with accession number PRJEB121486. The custom *Edwardsiella* spectra database for the Bruker MALDI-TOF is available on request from the authors.

## Supplementary Materials

The following supporting information can be downloaded at https://www.mdpi.com/article/10.3390/pathogens15090911/s1: Table S1: Clinical signs associated with *Edwardsiella* isolates; Table S2: *Edwardsiella* sp. genomic and annotation characteristics; Table S3: Biochemical analysis using API20E identification system.
