# Supplementary material for: Characterization of Edwardsiella Strains Isolated from Fish in The Netherlands (1995–2020) Shows a Relationship Between Edwardsiella Species and Source
Source: Pathogens. 2026 Aug 29;15(9):911. doi: 10.3390/pathogens15090911 (PMC13610115; doi:10.3390/pathogens15090911)
Supplement: Supplementary file 1 [file pathogens-15-00911-s001.zip › Table S1.pdf]

Table S1. Clinical signs associated with the *Edwardsiella* isolates from this study.

Isolate ID	Host	Isolated from	Clinical signs
WBVR4020328	European eel	unknown	unknown
WBVR4019560	European eel	unknown	skin ulcers, increased mortality
WBVR6012578	European eel	unknown	unknown
WBVR403175	European eel	spleen, liver, and kidney	skin hemorrhages, internal hemorrhages, increased mortality
WBVR421218	European eel	internal organs	pale animals, bulging eyes, internal hemorrhages, increased mortality, mixed infection with EVE[1]
WBVR509171	European eel	skin, liver, and kidney	pale animals, skin hemorrhages, skin lesions, internal hemorrhages, increased mortality
WBVR524362	Malawi cichlid	liver and kidney	labored breathing, ascites, pale liver and kidney, increased mortality
WBVR560693	channel catfish	skin, liver, and kidney	ascites, pale liver and kidney, increased mortality
WBVR618406	Tanganyika cichlid	abdominal fluid	bulging eyes, ascites, increased mortality
WBVR625534	European eel	liver	skin hemorrhages, skin lesions, increased mortality
WBVR4020328	European eel	skin lesions	skin hemorrhages, skin lesions, increased mortality
WBVR4019560	European eel	skin, liver, and kidney	skin hemorrhages, skin lesions, pale liver, brownish kidneys, increased mortality
WBVR6012578	European eel	skin and internal organs	skin hemorrhages, skin lesions, enlarged spleen, discoloration spleen and liver, increased mortality
WBVR6032203	African catfish	kidney	skin hemorrhages, skin lesions, pale liver, enlarged kidneys, increased mortality
WBVR6033894	discus fish	liver	skin lesions, reduced feed intake
WBVR7020313	European eel	skin and internal organs	ascites, discolored liver, pale kidney, increased mortality, mixed infection with *Vibrio vulnificus*
WBVR9008671	common sole	internal organs	pale spleen and kidney, brownish liver, slightly increased mortality
WBVR9009703	European eel	skin lesions and spleen	bloody gills, skin hemorrhages, increased mortality, mixed infection with EVE[1] and EVEX[2]
WBVR9010264	European eel	eye	eye lesions
WBVR9011798	European eel	internal organs	none, screening animal health status
WBVR9019448	common sole	kidney	pale liver and kidney, increased mortality
WBVR10001048	common sole	liver and kidney	skin lesions, pale spleen, liver, and kidney, increased mortality
WBVR10006632	electric eel	internal organs	skin lesions, ascites, pale kidney, increased mortality
WBVR10013402	European eel	liver	skin hemorrhages, skin lesions, increased mortality, mixed infection with AngHV1[3] and EVEX[2]

WBVR10015091	European eel	internal organs	skin hemorrhages, skin lesions, increased mortality, mixed infection with AngHV1[3] and EVEX[2]
WBVR11019050	European eel	internal organs	pale gills, discoloration skin, slightly increased mortality
WBVR12001944	European eel	skin and internal organs	skin lesions
WBVR14008484	turbot	internal organs	skin hemorrhages, skin lesions, ascites, pale spleen, liver, and kidney, red intestine, increased mortality, mixed infection with *Vibrio* spp.
WBVR14008838	European eel	skin lesions	skin lesions, mixed infection with AngHV1[3]
WBVR15015646	electric eel	electric organs	deep-red gills, skin discoloration due to parasitic infection, pale and enlarged liver, increased mortality
WBVR16005880	electric eel	skin and internal organs	skin hemorrhages, skin lesions, increased mortality
WBVR17005947	European eel	skin lesions and liver	skin lesions, red head, increased mortality
WBVR19008011	European eel	skin and internal organs	skin hemorrhages, skin lesions, enlarged spleen and kidney, discoloration liver, increased mortality
WBVR20001316	freshwater angelfish	internal organs	unknown
WBVR20007784	European eel	internal organs	deep-red gills, skin hemorrhages, skin lesions, enlarged spleen, discoloration liver, increased mortality
WBVR20016336	koi carp	internal organs	bulging eyes, eye and skin lesions, discoloration liver, pale kidney, reduced feed intake
WBVRX23018022	N.A.[4]	N.A.	N.A.

[1] EVE: Eel virus European; [2] EVEX: Eel virus European X; [3] AngHV1: Anguillid herpesvirus 1; [4] N.A., from water from RAS system with African catfish
