# Supplementary material for: Characterization of Edwardsiella Strains Isolated from Fish in The Netherlands (1995–2020) Shows a Relationship Between Edwardsiella Species and Source
Source: Pathogens. 2026 Aug 29;15(9):911. doi: 10.3390/pathogens15090911 (PMC13610115; doi:10.3390/pathogens15090911)
Supplement: Supplementary file 1 [file pathogens-15-00911-s001.zip › Table S2_AAK.pdf]

Supplementary materials

Table S2. *Edwardsiella* spp. chromosome 1 genomic and annotation characteristics based on Bakta version1.11.4 and full database v6.0. *Length*: total assembled sequence length of chromosome 1; *Count*: number of assembled contigs or scaffolds; *GC*: percentage of guanine (G) and cytosine (C) nucleotides in the assembled genome; *N50*: length of the shortest contig/scaffold among the longest sequences that together comprise at least 50% of the total assembly length; *N90*: length of the shortest contig/scaffold among the longest sequences that together comprise at least 90% of the total assembly length; *N ratio*: proportion of ambiguous nucleotide positions (N) in the assembly; *Coding density*: proportion of the genome occupied by predicted protein-coding sequences; *tRNAs*: number of transfer RNA genes; *tmRNAs*: number of transfer-messenger RNA genes; *rRNAs*: number of ribosomal RNA genes; *ncRNAs*: number of non-coding RNA genes; *ncRNA regions*: number of predicted non-coding RNA-containing genomic regions; *CRISPR arrays*: number of clustered regularly interspaced short palindromic repeat arrays; *CDSs*: number of predicted protein-coding sequences; *pseudogenes*: number of predicted disrupted or non-functional gene copies; *hypotheticals*: number of coding sequences annotated as hypothetical proteins; *sORFs*: number of predicted small open reading frames; *gaps*: number of unresolved regions in the assembly; *oriCs*: number of predicted chromosomal origins of replication; *oriVs*: number of predicted plasmid vegetative origins of replication; *oriTs*: number of predicted origins of transfer involved in conjugative DNA transfer; and *Plasmids*: number of predicted extrachromosomal plasmid replicons.

	Edwardsiella sp.	*E. anguillarum*	*E. hoshinae*	*E. ictaluri*	*E. piscicida*	*E. tarda*
**Genome**						
Length	3481702	4200387	3759372	3833790	3761542	3651448
Count	1	1	1	1	1	1
GC	59.1	58.7	56.9	57.4	59.7	57.3
N50	3481702	4200387	3759372	3833790	3761542	3651448
N90	3481702	4200387	3759372	3833790	3761542	3651448
N ratio	0	0	0	0	0	0
Coding density	85.6	86	86.7	84.6	86.7	86.8
**Annotation**						
tRNAs	102	98	103	97	97	105
tmRNAs	1	1	1	1	1	1
rRNAs	28	22	25	25	25	32
ncRNAs	45	43	41	54	42	47
ncRNA regions	37	36	37	33	37	35
CRISPR arrays	2	2	0	0	0	0
CDSs	3008	3697	3343	3433	3268	3229
Pseudogenes	3	4	0	8	1	3
Hypotheticals	125	132	136	210	88	97
sORFs	4	4	3	2	5	4
Gaps	0	3	0	0	0	1
oriCs	1	1	1	1	1	1
oriVs	0	0	0	0	0	0
oriTs	0	0	0	0	0	0
**Plasmids**	2	2	1	2	1	2
