# Supplementary material for: Characterization of Edwardsiella Strains Isolated from Fish in The Netherlands (1995–2020) Shows a Relationship Between Edwardsiella Species and Source
Source: Pathogens. 2026 Aug 29;15(9):911. doi: 10.3390/pathogens15090911 (PMC13610115; doi:10.3390/pathogens15090911)
Supplement: Supplementary file 1 [file pathogens-15-00911-s001.zip › Table S3_JA.pdf]

Table S3. Biochemical analysis using API20E identification system

strain ID	*Edwardsiella* species	ONPG*	ADH	LDC	ODC	CIT	H2S	URE	TDA	IND	VP	GEL	GLU	MAN	INO	SOR	RHA	SAC	MEL	AMY	ARA	OX	7-digit profile number**	species identification	Confidence level
		1	2	4	1	2	4	1	2	4	1	2	4	1	2	4	1	2	4	1	2	4			
WBVR625534	*E. piscicida*	-	-	+	+	+	+	-	-	+	-	-	+	-	-	-	-	-	-	-	-	-	4744000	*E. tarda*	*99.4%*
WBVR12001944	*E. piscicida*	-	-	+	+	+	-	-	-	+	-	-	+	-	-	-	-	-	-	-	-	-	4344000	*E. tarda*	*99.4%*
WBVR9019448	*E. piscicida*	-	-	+	+	-	+	-	-	+	-	-	+	-	-	-	-	-	-	-	-	-	4544000	*E. tarda*	*99.9%*
WBVR9008671	*E. piscicida*	-	-	+	+	-	+	-	-	+	-	-	+	-	-	-	-	-	-	-	-	-	4544000	*E. tarda*	*99.9%*
WBVR17005947	*E. piscicida*	-	-	+	+	+	+	-	-	+	-	-	+	-	-	-	-	-	-	-	-	-	4744000	*E. tarda*	*99.4%*
Ea BF1	*E. anguillarum*	-	-	+	+	+	+	-	-	+	-	-	+	-	-	-	-	-	-	-	-	-	4744000	*E. tarda*	*99.4%*
WBVR524362	*E. tarda*	-	-	+	+	+	+	-	-	+	-	-	+	-	-	-	-	-	-	-	-	-	4744000	*E. tarda*	*99.4%*
WBVR618406	*E. tarda*	-	-	+	+	+	+	-	-	+	-	-	+	-	-	-	-	-	-	-	-	-	4744000	*E. tarda*	*99.4%*
WBVR6033894	*E. tarda*	-	-	+	+	+	+	-	-	+	-	-	+	-	-	-	-	-	-	-	+	-	4744002	*E. tarda*	*93.7%*
WBVR20001316	*E. tarda*	-	-	+	+	+	+	-	-	+	-	-	+	-	-	-	-	-	-	-	-	-	4744000	*E. tarda*	*99.4%*
WBVR6032203	*E. ictaluri*	-	-	+	-	-	-	-	-	-	-	-	+	-	-	-	-	-	-	-	-	-	4004000	*E. coli*	*52.7%*
WBVR560693	*E. ictaluri*	-	-	+	-	-	-	-	-	-	-	-	+	-	-	-	-	-	-	-	-	-	4004000	*E. coli*	*52.7%*
WBVR16005880	*E. ictaluri*	-	-	+	-	-	-	-	-	-	-	-	+	-	-	-	-	-	-	-	-	-	4004000	*E. coli*	*52.7%*
WBVR15015646	*E. sp.*	-	-	+	-	-	-	+	-	-	-	-	+	-	-	-	-	-	-	-	-	-	4014000	*P. damselae*	*55.7%*

*Abbreviations: ONPG: o-nitrophenyl-β-D-galactopyranoside (test for β-galactosidase); ADH**:** Arginine dihydrolase; LDC: Lysine decarboxylase; ODC: Ornithine decarboxylase; CIT: Citrate utilization; H2S: Hydrogen sulfide production; URE: Urease; TDA: Tryptophan deaminase; IND: Indole production; VP: Voges-Proskauer (acetoin production); GEL: Gelatinase (gelatin liquefaction); GLU: Glucose; MAN: Mannitol; INO: Inositol; SOR: Sorbitol; RHA: Rhamnose; SAC: Sucrose; MEL: Melibiose; AMY: Amygdalin; ARA: Arabinose; OX: Oxidase**A 7-digit profile number is obtained by adding up the scores for the positive tests in each of the seven triplets. The highest score possible for a triplet is 7 (1+2+4).
